# Determinants of Timing of Breastfeeding Initiation at Pumwani Maternity Hospital in Kenya: A Hospital‐Based Cross‐Sectional Study

**DOI:** 10.1155/bmri/8665550

**Published:** 2026-09-11

**Authors:** Michael Ofire Ofire, Susan Keino, Robert Ofwete, Alice Sipiyian Lakati

**Affiliations:** ^1^ Department of Epidemiology and Biomedical Statistics, Moi University, Eldoret, Kenya, mu.ac.ke; ^2^ Population Health and Environmental Department, Amref Health Africa, Nairobi, Kenya, amref.org; ^3^ Department of Health Management and Human Nutrition, Moi University, Eldoret, Kenya, mu.ac.ke; ^4^ Deputy Vice Chancellor, Research and Extension, Amref International University, Nairobi, Kenya

## Abstract

**Objective:**

To assess the determinants of the timing of breastfeeding initiation and analyze those that are associated with delayed breastfeeding initiation among mothers delivering at Pumwani Maternity Hospital, Nairobi, Kenya.

**Background:**

Breastfeeding initiation within the first hour of birth is vital because of the benefits of colostrum, which is highly concentrated in immunological and nutritional factors shortly after parturition. The 2014 KDHS revealed that approximately 62% of the sampled children were breastfed within the first hour of birth, with 60.8% in Nairobi County, falling short of the 2030 collective target of at least 70%.

**Design:**

This study employed a hospital‐based cross‐sectional design.

**Methods:**

A total of 363 postpartum women who had initiated breastfeeding at the time of the survey and had given birth within 24 h were consecutively recruited. Recruitment was conducted daily from the postnatal ward until the target sample size was reached. Data were collected using a structured, open‐ended questionnaire and analyzed in R Version 4.3.3. Both descriptive and inferential statistics were used. The chi‐square and Fisher′s exact tests were then used to assess the association between the grouping variables and the outcome of breastfeeding initiation. All significant factors were fitted in unadjusted binary logistic regression models and further in the multiple‐adjusted binary logistic regression model.

**Results:**

Of the 363 participants recruited into the study, 344 mothers were included in the final analysis because they could recall the timing of breastfeeding initiation. Among these mothers, more than half (201/344; 58.4%) initiated breastfeeding within the first hour of birth. Mothers who gave birth through the cesarean section (aOR: 7.92; 95% CI: 4.54–14.27, *p* < 0.001), had low birth weight (aOR: 2.97; 95% CI: 1.26–7.13, *p* = 0.013), and were without knowledge of the need to introduce a newborn to breast milk within the first hour of birth (aOR: 1.69; 95% CI: 1.06–8.86, *p* = 0.040) had significantly higher odds of breastfeeding initiation after the first hour of birth. Additionally, giving birth to more than one child (aOR: 3.77; 90% CI: 0.89–19.62, *p* = 0.083) and lack of support to initiate breastfeeding (aOR: 1.58; 90% CI: 0.96–2.61, *p* = 0.069) demonstrated suggestive evidence of an association with breastfeeding initiation after the first hour of birth but did not meet the study′s threshold for statistical significance at the 95% confidence level.

**Conclusion:**

The study′s findings highlight that although more than half (58.4%) of the mothers included in the final analysis had initiated breastfeeding within an hour of birth, this proportion still falls short of the World Health Organization′s collective target of achieving at least a 70% timely breastfeeding initiation rate by 2030. The study further identified several determinants associated with breastfeeding initiation after the first hour of birth. These include low birth weight (less than 2.5 kg), birth through the cesarean section, and the lack of prior knowledge among mothers on the importance of timely breastfeeding initiation. Additionally, giving birth to more than one child and healthcare workers failing to provide breastfeeding initiation support shortly after birth demonstrated suggestive evidence of an association with delayed breastfeeding initiation and may be considered important barriers to timely breastfeeding initiation within the first hour of birth.

**Relevance to Clinical Practice:**

The results of this study underscore the need for Pumwani Maternity Hospital and the County Government of Nairobi to conduct periodic training sessions for all hospital healthcare workers, equipping them with the necessary skills to strengthen adherence to the “Ten Steps to Successful Breastfeeding” outlined in the Baby‐Friendly Hospital Initiative. Further research is required to generate additional evidence that can improve breastfeeding practices.

**Impact Statement**

• Low rates of early breastfeeding initiation remain a global challenge and a notable problem in sub‐Saharan Africa, including Kenya.
• This study demonstrates that children born underweight (< 2.5 kg), those born by cesarean section, those whose mothers had incorrect information about the timing of breastfeeding initiation, those who gave birth to more than one child, and those who were not supported to breastfeed are the determinants of delayed breastfeeding initiation among mothers delivering at Pumwani Maternity Hospital, Nairobi, Kenya.
• Given that Pumwani Maternity Hospital serves a population beyond its catchment area and operates under the county government of Nairobi, the county government should establish systems to ensure comprehensive prenatal care, including mandatory monitoring of gestational weight gain and the provision of breastfeeding education during ANC visits. In addition, targeted support should be provided to mothers who have undergone cesarean section births or given birth to multiple babies to initiate breastfeeding within the first hour of birth. These measures will help improve compliance with the WHO recommendation under the “Ten Steps to Successful Breastfeeding” outlined in the Baby‐Friendly Hospital Initiative, which states that postnatal mothers should be encouraged to initiate breastfeeding within the first hour after birth.

## 1. Introduction

Breastfeeding promotion is considered a critical public health intervention with significant economic, health, and social benefits for countries aiming to achieve healthier, thriving populations [1]. In a bid to improve breastfeeding outcomes, the World Health Organization (WHO) and the United Nations Children′s Fund (UNICEF) developed the Baby‐Friendly Hospital Initiative (BFHI) global strategy in 1991 to protect, promote, and support breastfeeding practices in health facilities providing maternity and newborn services through the scale‐up implementation of the “Ten Steps to Successful Breastfeeding.” Specifically, Policy 4 of the BFHI underscores the need for healthcare workers involved in the birth process to support mothers initiating breastfeeding as soon as possible after birth, emphasizing kangaroo mother care [2]. Additionally, Policy 6 states that newborn babies should not be fed any foods or fluids other than breast milk unless medically indicated. Despite such policies, trends between 2014 and 2019, excluding China, indicate that only 49% of newborns worldwide were timely introduced into breast milk within the first hour of birth, and only 47% according to a 2022 UNICEF report [1–3].

The breastfeeding initiation rates varied across different regions from 2014 to 2019, with Eastern and Southern Africa (65%) recording the highest initiation rates, while the Middle East and North Africa recorded the lowest at 34%, whereas West and Central Africa averaged 46%, Latin America and the Caribbean 53%, East Asia and the Pacific 39%, South Asia 50%, and Eastern Europe and Central Asia 74% [4]. The varying trends were also observed by UNICEF (2022), which reported that Eastern Europe and Central Asia recorded 72%, South Asia 39%, and Eastern and Southern Africa 65% [4]. Similarly, in Kenya, according to the 2022 Kenya Demographic and Health Survey (KDHS) report, the average national breastfeeding initiation rate was suboptimal at 60.1%, despite the report not providing regional initiation rates like the 2014 KDHS report, which highlighted varying regional breastfeeding initiation rates across Kenya, ranging from 48.1% in Central Kenya, 80.8% in Northeastern Kenya, 60.8% in Nairobi, 52.8% in Western, 58.4% in Nyanza, 69.4% in Rift Valley, 64.9% in Eastern, and 62.1% in Coast [5, 6].

The variation persists in Kenya despite the designation of various health facilities, such as Pumwani Maternity Hospital (PMH), Kenyatta National Hospital, Moi Teaching and Referral Hospital, and county and private facilities, as baby‐friendly. Furthermore, the country has enacted policy recommendations under the Standards for Maternal Care and the National Policy on Maternal, Infant and Young Child Nutrition, which underscore the importance of early breastfeeding initiation within an hour of birth unless the Apgar score is low (< 7). To understand the regional disparity in breastfeeding initiation rates, it is necessary to assess the determinants of breastfeeding initiation within 1 h of birth to generate broader systemic and social knowledge that may help address barriers to timely breastfeeding initiation in Kenya. Addressing these barriers will ensure that newborns receive colostrum‐rich milk containing both immune and nonimmune factors. This is because delayed breastfeeding initiation after the first hour of birth is associated with higher risks of neonatal infections and mortality, with infants first breastfed an hour after birth and within the first day of life having a 33% greater risk of neonatal mortality than those who are timely introduced to breast milk [7].

The findings of this hospital‐based cross‐sectional study, which was conducted at PMH in Nairobi, Kenya, highlight the determinants of timely breastfeeding initiation that, if addressed, will increase the likelihood of achieving the 2030 collective target of ensuring that at least 70% of immediate postpartum mothers initiate breastfeeding within the first hour of birth. The study applied a consecutive sampling technique to recruit 363 postnatal mothers in the postnatal ward who had undergone a cesarean section or spontaneous vaginal delivery (SVD) and had reportedly initiated breastfeeding at the time of the survey. The study′s main objective was to assess the determinants of breastfeeding initiation within the first hour of birth and identify those that may increase the likelihood of initiating breastfeeding after the first hour. Identifying these factors will lead to the establishment of systems and structures that will help improve early breastfeeding initiation rates in Kenya.

## 2. Methods

This was a hospital‐based cross‐sectional study conducted in the postnatal ward of PMH, the largest maternity hospital in sub‐Saharan Africa and the third busiest in Africa, providing maternity services to mothers in Nairobi and adjoining counties [8]. The study involved postnatal mothers and their newborns who gave birth through spontaneous vaginal birth (SVB) and cesarean section at PMH between August 23, 2022, and September 6, 2022.

A consecutive sampling technique was employed to recruit mothers in the postnatal ward who met the inclusion criteria: had delivered at PMH within 24 h, regardless of birth modality, and agreed to participate in the research. Study participants were recruited daily from the first day of data collection until the target sample size was reached. After consent, their health records were reviewed before the interview to ensure that only mothers who delivered at PMH were enrolled in the study. Interviews were conducted in private by the principal investigator and the two trained research assistants, at the discretion of the study participants, to ensure confidentiality.

Data were collected electronically using mobile phones programmed with a digital version of the tool, which was loaded into CommCare and later exported to Excel for exploration and analysis in R Version 4.3.3. Descriptive analysis was conducted by generating a 2 × 2 table of summary statistics, highlighting the frequency of each variable for those who delivered within 24 h and those who did not. Chi‐square test for association and Fisher′s exact tests were used for hypothesis testing, with the latter applied to variables with expected counts of less than five in at least one of the cells. All significant factors (*p* value ≤ 0.05) were initially included in simple (unadjusted) binary logistic regression models and, for those that remained significant, subsequently in multiple‐adjusted models. The model was assessed for assumptions, including the absence of multicollinearity, the absence of influential values, and the normality of residuals. Model performance was evaluated using both the area under the receiver operating characteristic (AUROC) and by assessing the performance of true negatives and true positives. The study assumed a 5% level of significance. However, some variables with clinical relevance and/or *p* values between 0.05 and 0.10 in the bivariate analysis were included in the multiple‐adjusted binary logistic regression model and reported and interpreted as providing borderline evidence of association, given their potential clinical and public health importance, as supported by the existing literature.

The study was approved by Moi University′s Institutional Research and Ethics Committee under Approval Number: 0003962 and authorized by the National Commission for Science, Technology and Innovation under License Number: NACOSTI/P/21/13338. Additional approvals were obtained from the Nairobi Metropolitan Services Research Committee and the PMH Ethics and Review Committee before data collection.

## 3. Results

As shown in Table 1, a total of 363 mothers aged between 17 and 45 years, with a mean of 27.0 years and a standard deviation of 5.9 years, 27.0 (5.9; 17–45) were enrolled into the study. Most of them were married, 275 (75.8%), whereas only one was widowed, 1 (0.3%). Most participants′ highest level of formal education was high school 170 (46.8%), whereas only 8 (2.2%) had no formal education. Most participants, 356 (98.1%), lived in Nairobi County, and only 7 (1.9%) lived outside Nairobi. The primary source of income was through support from their husbands, 183 (50.4%). Most were self‐employed, 103 (28.4%), with a mean MUAC measurement of 27.7 cm and standard deviation of 3.6 cm, 27.7 (3.6; 20.1–45.2). Most surveyed mothers were Christians, 335 (92.3%), whereas the rest were Muslims. Those that were primiparous were 131 (36.1%), whereas the rest were multiparous; 350 (96.4%) gave birth to a single neonate, whereas the rest gave birth to twins. A bigger percentage of the mothers gave birth through SVB, 258 (71.1%), with a mean gestational age of 38.7 months and standard deviation of 2 months, 38.7 (2.0; 29–43).

**Table 1:** Maternal sociodemographic and socioeconomic characteristics.

Variable	Number (%)
Age	
Mean age (SD; min–max)	27.0 (5.9; 17–45)
Residency	
Nairobi	356 (98.1)
Outside Nairobi	7 (1.9)
Marital status	
Married	275 (75.8)
Never married	72 (19.8)
Separated	4 (1.1)
Divorced	5 (1.4)
Widowed	1 (0.3)
Cohabiting	6 (1.7)
Religion	
Christian	335 (92.3)
Muslim	28 (7.7)
Level of education	
No formal education	8 (2.2)
Primary	108 (29.8)
Secondary	170 (46.8)
College	65 (17.9)
University	12 (3.3)
Occupation	
Formal employment	39 (10.7)
Casual	67 (18.5)
Housewife	65 (17.9)
Student	14 (3.9)
Unemployed	75 (20.7)
Self‐employed	103 (28.4)
Primary source of income	
Salary job	73 (20.1)
Own business	68 (18.7)
Husband	183 (50.4)
Other	39 (10.7)
Maternal nutrition status	
Maternal nutritional status: mean MUAC (SD; min–max)	27.7 (3.6; 20.1–45.2)
If child is first born child	
No	232 (63.9)
Yes	131(36.1)
Number of children besides the newborn	
1	102 (44.0)
2	73 (31.5)
3	40 (17.2)
4	13 (5.6)
5	1 (0.4)
6	3 (1.3)
Number of children born	
1	350 (96.4)
2	13 (3.6)
Gestational age at birth	
Mean gestational age (SD; min–max)	38.7 (2.0; 29‐43)
Birth modality	
SVB	258 (71.1)
CS	105 (28.9)
Attended ANC (%)	
No	5 (1.4)
Yes	358 (98.6)
Received information on breastfeeding initiation and its importance (%)	
No	164 (45.2)
Yes	199 (54.8)
Source of breastfeeding information (*n* = 199) (%)	
PMH	90 (45.2)
Other hospital or health center	99 (49.8)
Traditional birth attendant	1 (0.5)
Healthcare worker	1 (0.5)
MCH booklet	3 (1.5)
Other	5 (2.5)
Meal you should first give to your newborn (%)	
Breast milk	361(99.5)
Breast milk and formula	2 (0.6)
How long after birth a child should be introduced to breast milk (*n* = 361, two did not respond) (%)	
Immediately	155 (42.9)
Within an hour	68 (18.8)
After an hour	21 (5.8)
Do not know	117 (32.4)
If child is first born child	
No	232 (63.9)
Yes	131 (36.1)
Number of children born in a single birth (%)	
1	350 (96.4)
2	13 (3.6)
Where the mother first breastfed from (%)	
Theater	55 (15.2)
Maternity room	222 (61.2)
Postnatal ward	36 (9.9)
NBU	34 (9.4)
Other	16 (4.4)

A majority of the surveyed participants had attended ANC, 358 (98.6%), and received information on breastfeeding initiation and its importance, 199 (54.8%). Mothers who had received breastfeeding information from other hospitals or health centers were 99 (49.8%), followed by PMH, 90 (45.2%), with slightly over half, 114 (57.3%), receiving breastfeeding counseling before delivery during ANC visits. Most of the mothers had prior knowledge of the first meal a newborn should be given, breast milk, 361 (99.5%), and on how long after birth a child should be introduced to breast milk; immediately, 155 (42.9%), and within an hour, 68 (18.8%). Most of them had not fed the baby anything besides breast milk since birth, 332 (91.5%). For most mothers, the child delivered was not their firstborn child, 232 (63.9%). Most of the surveyed mothers had given birth to one child in a single birth, 350 (96.4%). Whereas in the hospital, most mothers first breastfed from the delivery room, 222 (61.2%).

Most newborns had an Apgar score of 7+, 351 (96.7%), and most were males 197 (54.3%). Most newborns had not been fed on anything other than breast milk since birth, 332 (91.5%), with 25 (80.7%) fed on infant formula among those that were given additional feeds beyond breast milk (Table 2).

**Table 2:** Characteristics of newborn babies.

Variable	Number (%)
Sex of child	
Male	197 (54.3)
Female	166 (45.7)
Apgar score (%)	
4–6	12 (3.3)
7+	351 (96.7)
Baby fed on anything else other than breast milk since birth (%)	
No	332 (91.5)
Yes	31 (8.5)
What the baby was given (%)	
Infant formula	25 (80.7)
iv fluids	6 (19.4)

### 3.1. Timing of Breastfeeding Initiation Among Mothers Delivering at PMH, Nairobi, Kenya

**Figure 1:**
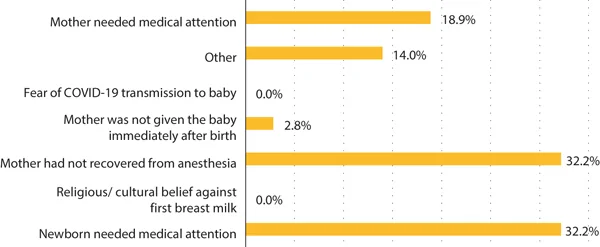
Reasons for late breastfeeding initiation among mothers giving birth at Pumwani Maternity Hospital, Nairobi County, Kenya.

Of the 363 postnatal mothers enrolled in the study (Table 1), 19 could not recall the time of their breastfeeding initiation and were therefore not included in the subsequent analysis assessing the determinants of the timing of breastfeeding initiation among the study participants, meaning that 344 mothers who delivered at PMH were included in the final analyses. From the 344 mothers, 201 (58.4%, 95% CI: 53.0–63.7) initiated breastfeeding timely, whereas the remaining 143 (41.6%, 95% CI: 36.3–47.0) began breastfeeding after an hour of birth (Table 3). Most participants who did not initiate breastfeeding within the recommended first hour of delivery indicated that the mother had not recovered from anesthesia (32.2%), the newborn child needed medical attention (32.2%), the mother needed medical attention (18.9%), and the mother was not given the baby (for rooming‐in) immediately after birth (Figure 1). Other reasons classified as “other” in Figure 1 included postpartum hemorrhage (*n* = 2), placenta retention (*n* = 2), mother was still undergoing a medical procedure (*n* = 1), mother lost consciousness (*n* = 1), and mother was in pain (*n* = 1).

**Table 3:** Breastfeeding initiation outcomes among mothers giving birth at Pumwani Maternity Hospital, Nairobi County, Kenya.

Time of breastfeeding initiation	Number (%) at 95% CI
Within the first hour of birth	201 (58.4%) 95% CI: 53.0–63.7
After an hour of birth	143 (41.6%) 95% CI: 36.3–47.0

### 3.2. Determinants Associated With the Timing of Breastfeeding Initiation Among Mothers Delivering at PMH, Nairobi, Kenya

The bivariate analysis conducted through the Pearson chi‐square test or Fisher′s exact test established that there is a significant association between the timing of breastfeeding initiation and whether the postnatal mother received support to initiate breastfeeding (*p* = 0.045), maternal knowledge on the recommended time to initiate breastfeeding (*p* = 0.002), the number of children born—either singleton birth or more (*p* = 0.019), gestational age (*p* = 0.007), and mode of delivery (*p* ≤ 0.001), as shown in Table 4. Furthermore, a significant association between the timing of breastfeeding initiation and both the neonate′s Apgar score (*p* ≤ 0.001) and birth weight (*p* ≤ 0.001) was observed. However, our study revealed that other determinants such as maternal residence (P= >0.999), marital status (*p* = 0.803), religion (*p* = 0.874), maternal level of education (*p* = 0.884), occupation of the mother (*p* = 0.310), maternal source of income (*p* = 0.787), age of the mother (*p* = 0.540), attendance to antenatal care clinics (*p* = 0.196), spouse accompanying to antenatal care clinics (*p* = 0.677), breastfeeding counseling during antenatal care clinics (*p* = 0.547), whether newborn was first child (*p* = 0.992), maternal nutrition status (*p* = 0.759), and number of children besides newborn (*p* = 0.922) were not significantly associated with the timing of breastfeeding initiation.

**Table 4:** Determinants associated with the timing of breastfeeding initiation among mothers giving birth at Pumwani Maternity Hospital, Nairobi, Kenya.

Variable	Overall *N* = 344	Initiated within an hour (*n* = 201)	Initiated after 1 h (*n* = 143)	Statistical test	Test statistics	*p*
Residence				Fisher′s exact		> 0.999
Nairobi	338 (98%)	197 (98%)	141 (99%)			
Outside Nairobi	6 (1.7%)	4 (2.0%)	2 (1.4%)			
Marital status				Chi‐square	0.440	0.803
Never married	64 (19%)	37 (18%)	27 (19%)			
Married	265 (77%)	154 (77%)	111 (78%)			
Other	15 (4.4%)	10 (5.0%)	5 (3.5%)			
Religion				Chi‐square	0.025	0.874
Christian	322 (94%)	189 (94%)	133 (93%)			
Muslim	22 (6.4%)	12 (6.0%)	10 (7.0%)			
Maternal level of education				Chi‐square	0.656	0.884
At least primary level education	108 (31.4%)	64 (31.8%)	44 (30.8%)			
Secondary	163 (47%)	96 (48%)	67 (47%)			
Tertiary	73 (21%)	41 (20%)	32 (22%)			
Occupation				Chi‐square	1.038	0.310
Unemployed	147 (43%)	91 (45%)	56 (39%)			
Employed	197 (57%)	110 (55%)	87 (61%)			
Source of income				Chi‐square	1.059	0.787
Salary job	67 (19%)	36 (18%)	31 (22%)			
Own business	67 (19%)	38 (19%)	29 (20%)			
Husband	174 (51%)	105 (52%)	69 (48%)			
Other	36 (10%)	22 (11%)	14 (9.8%)			
Maternal age				Chi‐square	1.234	0.540
19–23	131 (38%)	81 (40%)	50 (35%)			
24–35	177 (51%)	101 (50%)	76 (53%)			
Above 35	36 (10%)	19 (9.5%)	17 (12%)			
Support to initiate breastfeeding				Chi‐square	4.025	0.045 ^∗^
Yes	170 (49%)	109 (54%)	61 (43%)			
No	174 (51%)	92 (46%)	82 (57%)			
Attended ANC				Fisher′s exact		0.196
Yes	339 (99%)	200 (100%)	139 (97%)			
No	5 (1.5%)	1 (0.5%)	4 (2.8%)			
Spouses accompany to ANC				Chi‐square	0.173	0.677
Yes	103 (30%)	63 (32%)	40 (29%)			
No	236 (70%)	137 (69%)	99 (71%)			
Breastfeeding counseling during ANC				Chi‐square	0.362	0.547
No	151 (44%)	85 (42%)	66 (46%)			
Yes	193 (56%)	116 (58%)	77 (54%)			
Knowledge on introduction to breast milk				Chi‐square	9.482	0.002 ^∗∗^
Within an hour	219 (64%)	142 (71%)	77 (54%)			
After an hour/I do not know	125 (36%)	59 (29%)	66 (46%)			
If newborn is firstborn child				Chi‐square	< 0.001	0.992
Yes	124 (36%)	73 (36%)	51 (36%)			
No	220 (64%)	128 (64%)	92 (64%)			
Number of children born in the single birth				Chi‐square	5.522	0.019 ^∗^
One	331 (96%)	198 (99%)	133 (93%)			
More than one	13 (3.8%)	3 (1.5%)	10 (7.0%)			
Gestational age				Chi‐square	7.252	0.007 ^∗∗∗^
≥ 37 weeks (ref)	308 (90%)	188 (94%)	120 (84%)			
< 37 weeks	36 (10%)	13 (6.5%)	23 (16%)			
Birth modality				Chi‐square	57.570	< 0.001 ^∗∗∗^
SVB	249 (72%)	177 (88%)	72 (50%)			
CS	95 (28%)	24 (12%)	71 (50%)			
Apgar score				Fisher′s exact		< 0.001 ^∗∗∗^
7+	332 (97%)	201 (100%)	131 (92%)			
< 7	12 (3.5%)	0 (0%)	12 (8.4%)			
Maternal nutrition status				Chi‐square	0.094	0.759
MAM (19–23 cm)	47 (14%)	26 (13%)	21 (15%)			
Normal (> 23 cm)	297 (86%)	175 (87%)	122 (85%)			
Birth weight				Chi‐square	10.140	< 0.001 ^∗∗∗^
≥ 2.5 kg	301 (88%)	186 (93%)	115 (80%)			
< 2.5 kg	43 (13%)	15 (7.5%)	28 (20%)			
Number of children besides newborn				Chi‐square	0.010	0.922
More than one	124 (56%)	73 (57%)	51 (55%)			
One	96 (44%)	55 (43%)	41 (45%)			

Note: *p*– values were calculated using the Pearson chi‐square test or Fisher’s exact test. A *p*‐value < 0.05 marked with * was considered statistically significant. Variables marked by ** demonstrated a highly significant association. Variables marked with *** were considered significant at *p*‐value < 001.

### 3.3. Determinants Associated With Delayed Breastfeeding Initiation Among Mothers Delivering at PMH, Nairobi, Kenya

Knowledge of the introduction to breast milk. The odds for late initiation of breastfeeding were 2.99 times higher among those who did not know or did not have knowledge of whether they should have initiated breastfeeding within an hour of delivery (model estimate: 2.99; 95% confidence interval: 1.20–7.87; *p* value: 0.020) compared with those who had prior knowledge that they should initiate breastfeeding within 1 h.

Number of children. The odds of late breastfeeding initiation were 4.96 times higher among mothers who delivered more than one baby (model estimate: 4.96; 95% confidence interval: 1.49–22.44; *p* value: 0.016) compared with those who delivered one baby (singleton births).

Birth modality. There was a high likelihood of late initiation of breastfeeding after 1 h of delivery among mothers who delivered through the cesarean section compared with SVD. The odds were 7.27 times higher, with a 95% confidence interval ranging from 4.30 to 12.67 and a *p* value of less than 0.001.

Birth weight. The odds of late initiation of breastfeeding were 2.42 times higher (model estimate: 2.42; 95% confidence interval: 1.57–5.03; *p* value: 0.014) among those who delivered babies weighing less than 2.5 kg compared with those who delivered babies weighing at least 2.5 kg.

Gestation age. In the unadjusted model, the gestation age showed that higher odds of late breastfeeding initiation were 2.77 times higher among the mothers who delivered before 37 weeks of pregnancy (odds ratio: 2.77; 95% confidence interval: 1.37–5.83; *p* value: 0.005) compared with the mothers who carried the pregnancy for at least 37 weeks.

Support to initiate breastfeeding. Among the study participants, there was a 59% decrease in the odds of late initiation of breastfeeding (after an hour of birth) among mothers who were not supported in initiating breastfeeding in a timely manner, compared with those who received support. The estimate was 1.59 with a 95% confidence interval of between 1.04 and 2.46, with a significant *p* value of 0.035.

Although Apgar was significantly associated with the timing of breastfeeding initiation, it was excluded from the bivariate analysis because of the uneven distribution of observations across categories, which would have produced unreliable estimates.

Maternal nutrition status was included in the bivariate analysis because of its clinical relevance in influencing a mother′s ability to initiate breastfeeding within the first hour of birth based on existing literature. However, our study did not demonstrate any significant relationship between maternal nutrition status and the timing of breastfeeding initiation. As such, we recommend further investigation of its effect on the timing of breastfeeding initiation in larger studies.

After fitting variables into the adjusted model, three variables were significant: knowledge of breast milk introduction, birth weight, and birth modality after adjusting for birth modality, gestation age, number of children delivered in a single birth, and support to initiate breastfeeding.

Mothers without knowledge of the need to introduce a newborn to breast milk within the first hour of birth had 1.69 times more odds of initiating breastfeeding after 1 h of birth compared with those who knew about the need to introduce a newborn to breast milk within the first hour of birth, with a 95% confidence interval of 1.06–8.86 (*p* value: 0. 040). The odds were comparable between the unadjusted and adjusted models, showing an odds ratio of 2.06.

Mothers who gave birth to babies weighing less than 2.5 kg equally had 2.97 times higher odds of initiating breastfeeding after the first hour of delivery compared with those who gave birth to babies weighing more than 2.5 kg (aOR: 2.97; 95% CI: 1.26–7.13, *p* value: 0.013). This was similar to the unadjusted odds, in which mothers who gave birth to babies weighing less than 2.5 kg were significantly associated with breastfeeding initiation after the first hour of birth (OR: 3.02, 95% CI: 1.57–6.03, *p* value < 0.001).

There were 7.92 times increased odds (95% confidence interval: 4.54–14.27; *p* value less than 0.001) of initiating breastfeeding after an hour among those who gave birth through cesarean section compared with those who gave birth normally. The covariate‐adjusted model yielded results similar to the unadjusted model, with an odds ratio of 7.27 (95% confidence interval: 4.30–12.7) and a *p* value < 0.001 for breastfeeding initiation after the first hour of birth.

In the adjusted model, lack of support for breastfeeding initiation was associated with increased odds of breastfeeding initiation after the first hour of birth, with odds of initiation of breastfeeding after 1 h being 1.58 times among those who did not receive support (aOR: 1.58; 95% CI: 0.96–2.61; *p* value: 0.069) and those who had given birth to more than one child having 3.77 times more odds of late initiation compared with those who had given birth to one child (aOR: 3.77; 95% CI: 0.89–19.62; *p* value: 0.083). Although the association for these two variables did not reach the conventional threshold for statistical significance (*p* < 0.05), the demonstrated borderline evidence of association (*p* values between 0.05 and 0.10) was considered clinically relevant.

**Figure 2:**
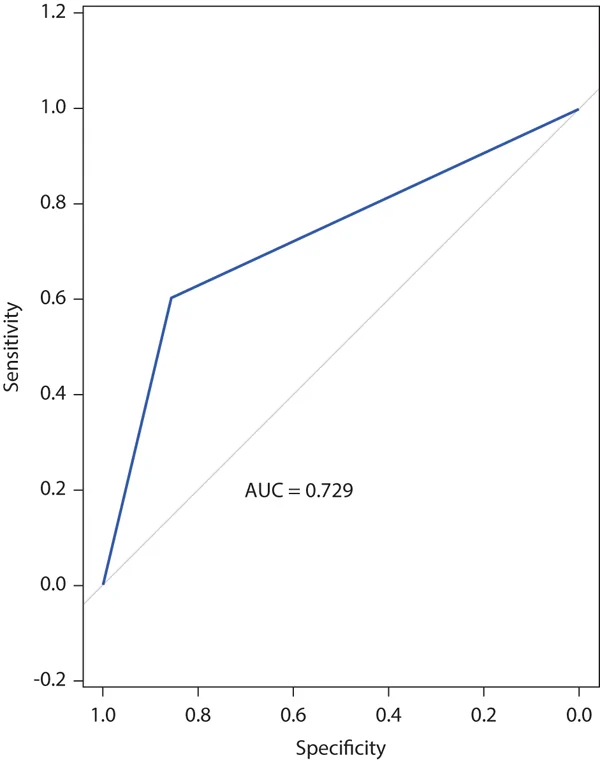
ROC curve for breastfeeding initiation model.

The AUROC analysis indicated that the model demonstrated acceptable discriminant performance in distinguishing mothers who initiated breastfeeding within the first hour of birth from those who initiated breastfeeding after the first hour, with an AUROC of 0.729 (Figure 2).

## 4. Discussion

Early breastfeeding initiation within the first hour of birth ensures that newborns derive maximum benefits from colostrum when its concentration levels and dosage are still high to provide the newborn with nutritional and immunological benefits [9–11]. Any delays in timely initiating breastfeeding increase the likelihood of the neonate missing out on colostrum‐rich milk, posing life‐threatening consequences [7].

Studies have demonstrated that many babies still wait longer than an hour after delivery to be introduced to breast milk, despite the various efforts to uphold the importance of breastfeeding initiation within the first hour of birth through the BFHI and maternal programs nested within the antenatal care. According to UNICEF Data on Breastfeeding (2022), the global breastfeeding initiation average was 47%, with Eastern Europe and Central Asia (72%) reporting almost double the initiation rates of South Asia at 39%, whereas the Eastern and Southern Africa region′s initiation rates stagnated at 65% as per data from the 2018 global report by UNICEF, “Capture the Moment ‐ Early Initiation of Breastfeeding: The Best Start for Every Newborn Capture [4–12].”

The rate of early initiation of breastfeeding in Kenya has not significantly changed over the years, from 58% in 1998 to 62% in 2014, and the latest 2022 KDHS indicates a slight drop to 60.1%. This is despite the latest Kenya Master Health Facility noting that all facilities at Level 2 and above provide maternity services. Levels 2 (dispensaries) and 3 (health centers) provide basic maternity services, including antenatal care, normal deliveries, and postnatal care, whereas Levels 4 and above provide comprehensive maternity services, including specialized care for complicated pregnancies and births through the cesarean section. With a vast network of health facilities providing maternity services and existing policies to strengthen and standardize maternal, infant, and young child care, it is expected that more mothers will receive comprehensive education and enabling support to initiate breastfeeding timely, thereby improving early breastfeeding initiation rates. Furthermore, the facility delivery rates have improved in Kenya over time because of friendly maternal policies to encourage more mothers to deliver from health facilities under the support of skilled birth attendants, who would then be expected to educate, create an enabling environment, and support postnatal mothers on immediate initiation of breastfeeding shortly after birth to improve the overall breastfeeding initiation rates [13].

Research conducted at PMH by Baya [14]partly aimed to identify the reasons for the delay in breastfeeding initiation and focused only on mothers who gave birth via CS. The researcher found that hospital factors primarily accounted for low initiation rates among CS mothers. However, with the study leaving out SVB mothers, gaps still exist in understanding the determinants of early initiation of breastfeeding with a pure focus on both SVB and CS mothers. As such, our study is aimed at bridging this knowledge gap and understanding the determinants associated with the timing of breastfeeding initiation among both SVB and CS mothers, which, if understood, may help the county government of Nairobi and other counties in Kenya establish systems and structures that can help more mothers initiate breastfeeding timely as recommended by WHO within the first hour of birth.

This study, conducted at PMH, established that only 58.4% (95% CI: 53.0–63.7) of the surveyed mothers included in the final analysis initiated breastfeeding within the first hour of birth (Table 3), and these findings are similar to the results of the 2022 KDHS surveys (60.1%). The initiation rates remain relatively low despite PMH being one of the pioneer facilities designated as baby‐friendly after the launch of the BFHI in Kenya in 1991 [15]. This demonstrates that compliance with the BFHI policy at the service level within the health facilities requires significant improvement to accelerate the achievement of the 2030 collective target of ensuring that at least 70% of newborns are initiated into breastfeeding within the first hour of birth [16]. The remaining surveyed mothers who did not initiate breastfeeding within the recommended first hour of delivery cited various reasons. These include the mother had not yet recovered from anesthesia, the newborn child needed medical attention, the mother needed medical attention, and the mother was not given the baby (for rooming‐in) immediately after birth, postpartum hemorrhage, placenta retention, the mother was still undergoing a medical procedure, the mother lost consciousness, and the mother was in pain (Figure 1).

Prenatal breastfeeding education has demonstrated increased success in improving the timing of breastfeeding initiation [17–23]. This is consistent with our findings, which established a significant relation between prior knowledge and introducing a newborn to breast milk within the first hour of birth. As such, the timely initiation of breastfeeding in our study may have been higher if the 77 mothers who recognized the importance of early breastfeeding initiation within an hour of birth had adhered to the prenatal breastfeeding education. However, our findings were inconsistent with those of Baya and Exavery et al., who found no significant relationship between prior knowledge of the importance of timely breastfeeding initiation and early breastfeeding initiation within an hour of birth [14, 18–23].

This study also found that mothers who delivered babies weighing less than 2.5 kg had significantly higher odds of initiating breastfeeding after the first hour of delivery than those who delivered babies weighing more than 2.5 kg (Tables 5 and 6). These findings are consistent with those of Senarath et al., Khanal et al., and Woldeamanuel [24–26], who also found that newborns with low birth weight were more likely to experience delayed breastfeeding initiation. This is explained by Patel et al., who assert that the delayed breastfeeding initiation among underweight babies can result from the babies′ poor suckling ability. Nonetheless, our findings are inconsistent with those of Ahinkorah et al., who found that breastfeeding initiation rates were higher among low‐birth‐weight babies than those with normal birth weight [14, 21–27].

**Table 5:** Determinants of breastfeeding initiation among mothers giving birth at Pumwani Maternity Hospital, Nairobi County, Kenya.

Unadjusted (univariable analysis)			
Variable	Odds ratio	95% CI	*p*
Support to initiate breastfeeding			
Yes (ref)	—	—	—
No	1.59	1.04–2.46	0.035 ^∗^
Knowledge on introduction to breast milk			
Within an hour (ref)	—	—	—
After an hour/I do not know	2.99	1.20–7.87	0.020 ^∗^
Number of children born in the single birth			
One	—	—	—
More than one	4.96	1.49–22.44	0.016 ^∗^
Birth Modality			
SVB (ref)	—	—	—
CS	7.27	4.30–12.67	< 0.001 ^∗∗∗^
Birth weight			
≥ 2.5 kg (ref)	—	—	
< 2.5 kg	2.42	1.57–5.03	0.014 ^∗^
Maternal nutritional status (MUAC)			
MAM (ref)			
Normal	0.86	0.47–1.62	0.642
Gestational age			
≥ 37 weeks (ref)			
< 37 weeks	2.77	1.37–5.83	0.005 ^∗∗^

*Note:* A *p* − value < 0.05 marked with * was considered statistically significant. Variables marked with ** were considered significant at *p* − value = 005. Variables marked with *** were considered significant at *p* – value <001.

**Table 6:** Multivariate analysis on determinants of breastfeeding initiation among mothers giving birth at Pumwani Maternity Hospital, Nairobi County.

Variable	Unadjusted			Adjusted (multivariable)		
	Odds ratio	95% CI	*p*	Odds ratio	95% CI	*p*
Support to initiate breastfeeding						
Yes (ref)	—	—	—	—	—	—
No	1.59	1.04–2.46	0.035 ^∗∗∗^	1.58	0.96–2.61	0.069^●^
Knowledge on introduction to breast milk						
Within an hour (ref)	—	—	—	—	—	—
After 1 h	2.06	1.32–3.24	0.002 ^∗∗∗^	1.69	1.06–8.86	0.040 ^∗^
Number of children delivered in the single birth						
One (ref)	—	—	—	—	—	—
More than one	4.96	1.49–22.40	0.016 ^∗∗∗^	3.77	0.89–19.62	0.083^●^
Gestation age						
*37* weeks and above (ref)	—	—	—	—	—	—
Less than 37 weeks	2.77	1.37–5.83	0.005 ^∗∗∗^	1.58	0.63–3.97	0.325
Birth weight						
≥ 2.5 kg (ref)	—	—	—	—	—	—
< 2.5 kg	3.02	1.57–6.03	0.001 ^∗∗∗^	2.97	1.26–7.13	0.013 ^∗^
Birth modality						
SVB (ref)	—	—	—	—	—	—
CS	7.27	4.30–12.70	< 0.001 ^∗∗∗^	7.92	4.54–14.27	< 0.001 ^∗∗∗^
Maternal nutritional status						
MAM (ref)	—	—	—	—	—	—
Normal	0.86	0.47–1.62	0.642	−0.63	0.31–1.32	0.216

*Note:* A *p* − value < 0.05 marked with * was considered statistically significant. Variables marked with *** were considered significant at *p* – value < 001. Variables with 0.05 ≤ *p* < 0.10 are marked with ^●^ and are reported as showing suggestive evidence of association due to their clinical relevance.

Several studies have demonstrated that birth modality is significantly associated with the timing of breastfeeding initiation. Specifically, mothers who give birth through cesarean section are more likely to initiate breastfeeding after an hour of birth compared with those who undergo SVBs [2]. This study found that 95 (28%) of the 344 surveyed mothers included in the final analysis gave birth through cesarean section. Of the 95, only 24 (25%) initiated breastfeeding within the first hour of birth (Table 4), indicating that approximately three‐quarters of the surveyed mothers who gave birth via cesarean section did not introduce their newborns to breast milk as recommended by WHO within the first hour of birth.

Among the mothers who delayed breastfeeding initiation following the cesarean section birth, only 32.2% of the mothers attributed the late initiation to delayed recovery from anesthesia (Figure 1). As such, this study demonstrates that other factors beyond postoperative recovery from anesthesia among CS mothers may contribute to delayed breastfeeding initiation after an hour of birth. These findings are consistent with Exavery et al. and Ahmed and Salih, who established that birth modality is significantly associated with the timing of breastfeeding initiation, with mothers who gave birth through cesarean section having lower odds of early initiation of breastfeeding compared to those born through SVBs [17, 18]. Similarly, our findings are consistent with the Ndirangu et al. [19] study in Namibia from demographic health survey data (2000–2013), Mekonen et al. [20] study in Ethiopia, and Ahinkorah et al. [21] study in Chad, which also established that the birth modality is significantly associated with the timing of breastfeeding initiation.

Despite delayed breastfeeding initiation being significantly associated with giving birth through the cesarean section, evidence suggests that timely breastfeeding initiation is still feasible among CS mothers when given appropriate support. This is because most cesarean sections are done under spinal anesthesia, with only a few done through general anesthesia, and as such, the mother is mostly alert and able to hold the infant shortly after birth, and this can lead to timely breastfeeding initiation from the theater [14, 24–30]. This evidence is also documented in a Lancet series by Rollins et al. [31], which established that in the presence of adequate support to initiate breastfeeding, a cesarean section birth was not independently associated with delayed breastfeeding initiation (risk ratio [RR] 0.95 [95% CI: 0·84–1·07]).

Consistent with this evidence, our study found that the odds of initiating breastfeeding after an hour of birth increased among mothers who were not supported to initiate breastfeeding (Tables 5 and 6). Even though these findings did not demonstrate the conventional 5% significance threshold in the adjusted analysis, the observed effect size and direction were consistent with similar studies and recommendations with Step 4 of the BFHI′s “Ten Steps to Successful Breastfeeding”, which emphasizes immediate skin‐to‐skin contact and support for breastfeeding initiation within an hour of birth [2]. Given its clinical relevance as outlined in Step 4 of the BFHI, this finding warrants additional investigation in larger studies. This is because strengthening adherence to breastfeeding initiation support by promoting immediate kangaroo mother care can be an important strategy to support early rooming‐in for mothers who gave birth through CS, which could encourage more mothers to initiate breastfeeding within the first hour of birth [30, 32, 33].

Our study also demonstrated an inverse relationship between timely breastfeeding initiation and the number of children born in a single birth, with mothers who delivered more than one baby being less likely to initiate breastfeeding within the first hour of birth (Tables 5 and 6). Although this association did not meet the conventional 5% significance threshold in the adjusted analysis (Table 6), the observed effect size and direction are consistent with other studies, which established that the odds of breastfeeding after an hour of birth are higher among mothers who gave birth to more than one child since they may have given birth via cesarean section [34]. As highlighted above, there is a significant association between delayed breastfeeding initiation and birth via CS. As such, mothers who delivered more than one baby can still benefit from breastfeeding support that should be provided shortly after birth, and this can encourage more mothers to initiate breastfeeding within the first hour of birth. Given the clinical relevance and existing literature on the association between the number of children born and the timing of breastfeeding initiation, there is a need to conduct further investigation in larger studies.

This study had several limitations. First, it relied on self‐reported data, which made it difficult to verify some of the collected data independently. Secondly, recall bias may have been a challenge regarding the exact time of breastfeeding initiation and breastfeeding information provided during ANC visits. Thirdly, due to the cross‐sectional study design, there is a temporal bias, because the data were collected within a specific period and may not capture changes in breastfeeding initiation practices over time. Fourth, selection bias because the study was conducted at a single facility, and participants may not be representative of all mothers in Kenya. However, although this may limit the representativeness of the sample and the generalizability of the findings to all mothers in Kenya, it is noteworthy that the prevalence of breastfeeding initiation within the first hour of birth observed in our study (58.4%; 95% CI: 53.0–63.7) was comparable with the national estimate reported in the 2022 KDHS (60.1%). Finally, although several potential confounders were adjusted for in the analysis, residual confounding due to unmeasured characteristics cannot be ruled out.

## 5. Conclusion

In conclusion, the study findings highlight that slightly more than half (58.4%) of the mothers included in the final analysis who had delivered at PMH, Nairobi, Kenya, initiated breastfeeding within the first hour of birth. This still falls short of the WHO collective target of at least 70% of timely breastfeeding initiation by 2030. Secondly, the results demonstrate a significant association between the timing of breastfeeding initiation and whether the postnatal mother received support to initiate breastfeeding, prior maternal knowledge on the proper timing for breastfeeding initiation, the number of children born, gestational age, mode of delivery, neonatal Apgar score, and birth weight among mothers delivering at PMH, Nairobi, Kenya. The study also demonstrates that children born underweight (< 2.5 kg), those born by cesarean section, and those whose mothers had incorrect information about the timing of breastfeeding initiation are determinants that are associated with breastfeeding initiation after the first hour of birth among mothers delivering at PMH, Nairobi, Kenya [35]. Additionally, giving birth to more than one child and healthcare workers failing to provide breastfeeding initiation support shortly after birth demonstrated suggestive evidence of an association with delayed breastfeeding initiation and may be considered important barriers to timely breastfeeding initiation within the first hour of birth. However, additional evidence on these two variables is needed in larger studies.

Based on our findings as presented in Table 1, 358 (98.6%) of the surveyed mothers attended ANC during pregnancy; however, only 199 (54.8%) reportedly received information on the importance of breastfeeding initiation within the first hour of birth, with nearly half, 99 (49.8%), receiving this information from other hospitals and health centers other than PMH. These findings demonstrate that a substantial proportion of women attend ANC in other facilities and only go to PMH to give birth.

Because PMH operates under the county government of Nairobi and serves a population transcending its immediate catchment area, the county government needs to establish systems to strengthen and standardize prenatal care across all health facilities in the county, as well as postnatal care in facilities that provide maternity services. This should include strengthening mandatory monitoring of gestational weight gain and breastfeeding education during ANC visits. In addition, providing targeted support to mothers who have given birth through cesarean section or given birth to multiple babies should be strengthened to help them initiate breastfeeding within the first hour of birth. These measures will help improve compliance with the WHO recommendation under the “Ten Steps to Successful Breastfeeding” outlined in the BFHI, which states that postnatal mothers should be encouraged and supported to initiate breastfeeding within the first hour after birth.

## Author Contributions

M.O.O. led the initial draft of the manuscript, whereas S.K., R.O., and A.S.L. contributed to the review and further revision. R.O. is a medical statistician.

## Funding

No funding was received for this manuscript.

## Disclosure

All authors provided final approval of the paper before submission. Service providers helped in identifying women who had delivered within 24 h at Pumwani Maternity Hospital during the survey period. This study adhered to the STROBE cross‐sectional reporting guidelines.

## Conflicts of Interest

The authors declare no conflicts of interest.

## Data Availability

The data that support the findings of this study are available on request from the corresponding author. The data are not publicly available due to privacy or ethical restrictions.
